# Linkage, case-control association, and family-based association tests for complex disorders

**DOI:** 10.1186/1753-6561-1-s1-s43

**Published:** 2007-12-18

**Authors:** Brian K Suarez, Robert Culverhouse, Carol H Jin, Anthony Hinrichs

**Affiliations:** 1 Department of Psychiatry, Washington University School of Medicine, 660 South Euclid, St. Louis, Missouri 63110, USA; 2 Department of Genetics, Washington University School of Medicine, 660 South Euclid, St. Louis, Missouri 63110, USA; 3 Department of Medicine, Washington University School of Medicine, 660 South Euclid, St. Louis, Missouri 63110, USA

## Abstract

We carried out an analysis of the Genetic Analysis Workshop 15 simulated Problem 3 data. We restricted ourselves to the present/absent phenotype. Linkage analysis revealed a very strong signal on chromosome 6. Association analysis revealed additional susceptible loci located on chromosomes 11 and 18. The latter two signals were subsequently verified with linkage analysis – but only after 20 replicates were pooled. Analysis of linkage disequilibrium patterns, in concert with family-based association tests, led us to infer the presence of a second chromosome 6 locus located in the vicinity of single-nucleotide polymorphisms 160–162. These analyses were carried out without knowledge of the model used to generate the simulation.

## Background

In the last few decades the genes responsible for hundreds of simple Mendelian phenotypes have been identified and their variants characterized. Progress on complex diseases, however, has been much slower. With the advent of new technologies capable of quickly genotyping millions of single-nucleotide polymorphisms (SNPs), the prospect of making substantial inroads in understanding complex phenotypes has improved. Nonetheless, to characterize the genetic architecture of complex phenotypes fully, a researcher will need to employ the full armamentarium of traditional methods, including linkage analysis, association analysis, and family-based transmission tests.

We chose to investigate the simulated Problem 3 data set. Briefly, the simulation was designed to mimic the familial pattern of rheumatoid arthritis (RA), including the effect of the DR ideotypes at the MHC on chromosome 6, a lifetime prevalence of 1.07%, a 3:1 female:male affection ratio, and a λ_s _of 9.03. A total of 100 replicates were simulated. Each replicate consisted of 1500 nuclear families with a pair of offspring with RA and a random sample of 2000 unrelated individuals each drawn from the offspring generation of families containing no RA cases. Further details can be found in Miller et al. [1].

## Methods

We performed linkage analysis on the affected sib-pair nuclear families. We restricted our attention for the preliminary analysis to the "sparse" (*N *= 9187) SNP map. The evidence for linkage was evaluated with MERLIN software [2]. Because we wanted to determine the consequence of linkage disequilibrium (LD), especially when parental genotypes are unavailable, we chose to compute the Kong and Cox [3] emendation of the PAIRS statistic [4]. The first replicate was analyzed in detail and, after association analysis was performed, the first 20 replicates were analyzed for selected chromosomes.

To evaluate the possibility of preferential transmission at the DRB1 locus, and for a subset of the SNPs "genotyped" for the whole-genome scan, we used FBAT [5] software.

We carried out an association analysis on the same sparse SNP map. From each of the first 50 replicates, we formed a group of unrelated cases (*N *= 1500) by selecting the most severely affected sib. If both sibs were equally affected, we selected the first sib. This case sample was compared to the N = 2000 controls supplied by the data providers. Ordinary chi-squares were computed for all SNPs (for both allele frequencies and genotype frequencies) in the sparse map.

To evaluate the consequences of LD, we used a program written by one of us (AH) that interfaces with the TRANSMIT package [6]. The software allows the user to select the value of D' and/or *R*^2 ^that will be used to thin the SNPs as well as the size of the base-pair window within which the LD evaluations are made. We chose a sliding window of 1 Mb and a LD level > 0.1 for *R*^2 ^for SNP thinning. Thus, all SNPs within one megabase of the first SNP were evaluated for LD. If SNP 1 was found to be in LD with SNP 2, say, then the SNP with the lowest heterozygosity was deleted. If SNP 1 was not deleted, then LD between SNP 1 and SNP 3 was evaluated, and so on. If SNP 1 was deleted, the edge of the window advances to SNP 2, etc.

## Results

**Figure 1:**
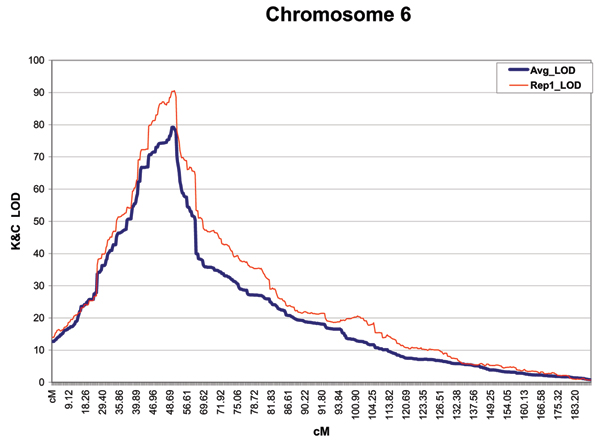
**LOD scores for chromosome 6 from Replicate 1 (thincurve)**. The average LOD (thick curve) was obtained from an analysis of the first 20 replicates.

Linkage analysis of Replicate 1 revealed a very strong signal on chromosome 6 and little else. Only one other chromosome attained a LOD score > 1.0 (SNP 8 on chromosome 20 had a nominal *p*-value of 0.003). By contrast, the linkage signal on chromosome 6 attained a maximum LOD of 90.33 at SNP 152. Moreover, the LOD scores were positive over the entire length of the chromosome. Figure 1 reports the LOD scores for chromosome 6, Replicate 1.

Table 1 reports the results of the association analysis for all case/control genotype comparisons that attained a *p*-value of 0.000005 for the same SNP on more than one replicate. This *p*-value ought to correct for the approximately 10,000 SNPs that were evaluated, although we hasten to note that because of the presence of LD, not all of these comparisons are independent. An interesting pattern on chromosome 6 was observed. There appears to be a cluster of significant comparisons proximal to the position where we obtained the highest LOD score. Thus at SNPs 138 and 139, all 50 replicates gave a significant association signal. The highest mean chi-square over Replicates 1–50 is found at SNP 153. The adjacent upstream SNP and the two adjacent down-stream SNPs also were significant in 50 out of 50 replicates. Table 1 also reports the presence of a highly significant association for SNP 389 on chromosome 11 and SNP 269 on chromosome 18.

**Table 1:** Summary of χ^2 ^genotype analysis (Replicates 1–50)

Chromosome	SNP	Frequency^a^	Mean χ^2^	Minimum χ^2^
6	128	34	31.00	13.63
	129	36	32.04	13.61
	130	40	36.99	14.49
	133	20	23.98	11.64
	134	49	43.38	21.71
	136	5	13.58	3.70
	137	9	17.43	1.49
	138	50	65.55	29.14
	139	50	65.55	28.88
	142	2	7.62	0.33
	144	3	14.02	2.22
	145	21	25.70	6.65
	147	42	39.62	15.82
	149	4	13.84	0.97
	150	45	42.25	15.62
	152	50	706.01	617.78
	153	50	1748.46	1613.22
	154	50	1545.92	1402.22
	155	50	322.80	260.67
	156	15	20.16	6.51
	160	49	52.33	24.27
	162	50	124.57	86.18
				
11	387	7	16.79	3.96
	389	50	120.65	81.29
				
18	269	44	35.99	20.38

^a^Frequency, number of replicates for which the analysis of genotypes resulted in a *p*-value < 0.05 after a Bonferroni correction for 10,000 tests.

**Figure 2:**
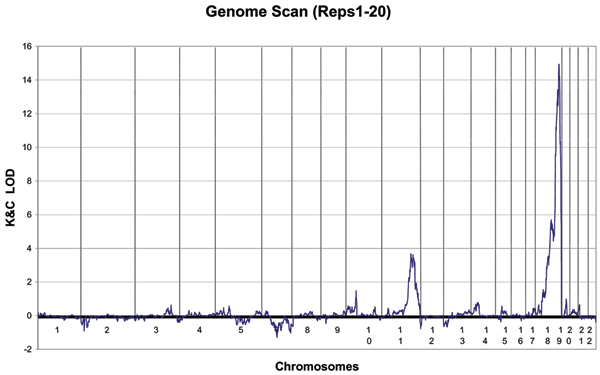
**Genome scan for all autosomes except chromosome6**. The LOD scores were computed after combining the first 20 replicates for a total of 30,000 affected sib pairs.

Because our linkage analysis of Replicate 1 revealed no evidence of linkage to either chromosome 11, SNP 389, or chromosome 18, SNP 269, (the LOD scores were 0.41 and 0.82, respectively), we decided to pool the first 20 replicates and repeat the analysis. Figure 2 reports the results for this second genome scan for all autosomes except chromosome 6. There is, indeed, a weak linkage signal on chromosome 11 at SNP 389 (the LOD score is 3.48) and a much stronger linkage signal on chromosome 18 at SNP 269 (the LOD score is 14.85).

LD analysis suggests that SNP 153 on chromosome 6 and HLA-DR locus are very close to one another and the effects attributed to SNP 153 are likely due to its nearly complete LD with HLA-DR (of the six possible haplotypes in Replicate 1 in cases and controls (i.e., 1_1, 1_2, 1_3, 2_1, 2_2, 2_3) the following counts were observed: 0, 114, 2525, 357, 0, 4 and 2, 293, 938, 2767, 0, 0, respectively).

The linkage analysis of chromosome 6 in the first replicate produced a massive signal. We were, nonetheless, surprised that for a chromosome that is modeled to have a gender-averaged genetic length of 197.5 cM, the LOD scores were positive at every SNP position. The case/control association analysis gave a hint that other susceptibility loci may be located under the large LOD peak show in Figure 1. In particular, the associations seen at SNPs 138/139 and at 160/162 appear separable from the major signal at SNP 153. Accordingly, we computed linkage disequilibrium statistics D' and R^2 ^coefficients between SNP 153 and the suspected proximal and distal association signal regions. We first estimated the haplotypes from the genotypes ignoring the phase information given by the data providers and then we used the phase information. When estimated from the genotypes of cases in Replicate 1, we found no compelling evidence of LD between SNP 153 and SNP 160 or 162 (D' and *R*^2 ^are 0.026, 0.0 and 0.093, 0.003, respectively). The phase-known haplotypes also give no evidence of LD. For the SNPs 138/139 vs. SNP 153 estimates, however, we find evidence of significant LD by both estimates (the phase-known chi-square is 31.4 and the phase-unknown maximum likelihood estimates yield a chi-square of 25.6).

**Figure 3:**
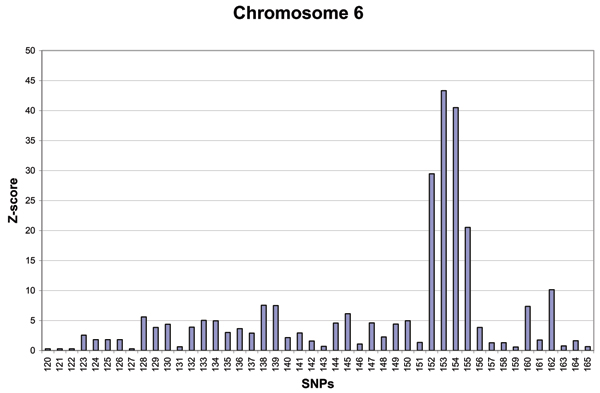
FBAT Z-scores for SNPs 120 to 165 of chromosome 6 of Replicate 1.

To further scrutinize the pattern of significant associations on chromosome 6, we performed an analysis of SNPs 120–165 with FBAT to determine whether there is preferential transmission at any of the sites that showed association. Figure 3 reports the results of this analysis. While many of the *Z*-scores are significant – especially SNPs 152–155 – it is noteworthy that the next four most deviant SNPs (138, 139, 160, and 162) are the same SNPs that gave a strong association signal.

This long-range LD – over a simulated distance of >3.7 MB – was unexpected and suggests that the association and FBAT signals at SNPs 138/139 are a consequence of the LD rather than an independent risk locus. Accordingly, if there is a second risk locus on chromosome 6, a location in the vicinity of SNPs 160–162 would seem to be favored.

To evaluate the influence of LD on the linkage analysis, we selectively removed SNPs so that all pairs within one Mb had a *R*^2 ^< 0.1. For chromosome 6 this involved the removal of 506 (75%) of the 674 SNPs. The resulting LOD distribution (not shown) is virtually identical to that produced with all 674 SNPs (the mean difference when evaluated at all 168 retained SNPs is 2.6 LOD units). This is not the case if parental genotypes are removed. For chromosome 6 using all 674 SNPs, but no parental genotypes, the linkage is still detected although the maximum LOD score is only 20.9 and is incorrectly positioned at SNP 121. The trimmed map (without parental genotypes) attains higher maximum LOD score (112.2) but is also incorrectly positioned at SNP 502.

## Discussion

It is instructive, and perhaps sobering, to realize that there are occasions in which linkage analysis is essentially powerless to detect a signal (in a sample of 30,000 affected sib pairs!) that is readily detected by association analysis in a sample approximately one-tenth the size. Cox and Bell [7] provided an example of a simple two-allele quasi-dominant model where the at-risk genotypes have low penetrance (and relatively low genotype frequency) that behaves in a manner similar to the region at or in the near vicinity of SNP 389 on chromosome 11.

The presence of LD is known to inflate linkage statistics and the problem is exacerbated when parental genotypes are unavailable [8]. Our analysis of the chromosome 6 data indicated that comparable LOD distributions were obtained with the full SNP map or the reduced SNP map as long as the parental genotypes were used. However, when parental genotypes are not used, neither the full nor the trimmed map produced consistent results. To determine if trimming could avoid making a type I error when parental genotypes are ignored or unavailable, we broke our blind and searched for a false linkage on the chromosomes where no genes were modeled. On chromosome 22, Replicate 1, we obtained a false positive signal at SNPs 11–13 with a LOD score of 7.16. We then applied our trimming algorithm, as described above, which resulted in the retention of 32 (43%) of the 75 SNPs. The maximum LOD score was 0.31 (at SNP 13) so no false inference would be drawn. The current version of Merlin requires haplotype clusters. The documentation reads: "Two limitations of the model are that it assumes no recombination within clusters and no linkage disequilibrium between clusters." Although this method is appropriate for many data sets, when dense markers are spaced uniformly across a chromosome, it is unclear how to define clusters and an alternative approach must be used.

Except to determine whether trimming SNPs in LD could decrease type I error in the absence of parental genotypes, as noted above, all of the work reported here was undertaken without knowledge of the generating model.

## Conclusion

True to what is known about rheumatoid arthritis, the major genetic effect appears to be due to HLA-DR alleles on chromosome 6. Three additional signals were detected in our blind analysis; a second chromosome 6 signal in the vicinity of SNP 160–162, a signal on chromosome 11 in the vicinity of SNP 389, and a signal on chromosome 18 in the vicinity of SNP 269. These latter two signals were best detected with association analysis. To be detected by linkage analysis, a prohibitively large sample size of families would be required.

## Competing interests

The author(s) declare that they have no competing interests.
